# ERRATUM

**DOI:** 10.1590/0074-02760230002er

**Published:** 2023-02-20

In the article “***Toxoplasma gondii*: a rapid method for the isolation of pure tachyzoites: preliminary characterization of its genome**”, DOI number: 10.1590/s0074-02761990000400007, published in *Mem Inst Oswaldo Cruz,* Rio de Janeiro, Vol. 85(4), 1990, on page 429:

Where it reads:

Viviana Pzsenny

It should read:

Viviana Pszenny

